# Bilateral Malrotation and a Congenital Pelvic Kidney with Varied Vasculature and Altered Hilar Anatomy

**DOI:** 10.1155/2015/848949

**Published:** 2015-11-10

**Authors:** J. Singh, N. Singh, K. Kapoor, M. Sharma

**Affiliations:** ^1^Department of Anatomy, Government Medical College & Hospital, Chandigarh, India; ^2^Department of Medicine, BPS, Government Medical College for Women, Khanpur Kalan, Sonepat, India; ^3^Department of Anatomy, Gian Sagar Medical College and Hospital, Patiala, India

## Abstract

Variations of structure and position of the kidney along with variations of renal vessels are most frequently reported. Rotational variations form a rare entity that are not cited in most embryology textbooks. During an educational cadaveric dissection of a 42-year-old male, a complex picture of bilateral anatomical variants was encountered. Malrotation of both kidneys and a left lobulated ectopic kidney along with open hilum was observed. The left kidney showed a pelvic position in front of sacral promontory with three renal arteries retaining its embryological aortoiliac branches and two renal veins draining into right common iliac vein. These variations have an embryological base. Pelvic kidney with rotational variation though comparatively rare assumes great importance in view of present-day surgical procedures like laparoscopic radical nephrectomy, percutaneous nephrectomy, and renal transplantation.

## 1. Introduction

The kidneys lie in the upper part of the paravertebral gutters, posterior to the peritoneum, tilted against the structures on the sides of the lowest two thoracic and upper three lumber vertebra, so that anterior and posterior surfaces face anterolaterally and posteromedially, respectively. In addition, the superior extremity of the right kidney lies at a lower level (eleventh intercostal space) than the left kidney (eleventh rib) because of the presence of liver. The inferior poles lie 2.5 cm above iliac crest [1]. Congenital anomalies of the urinary tract are often the underlying cause of several pathologies; 40% of these pathological conditions are due to variations in number, position, shape, and size of the kidney(s), calyces, ureter, or bladder [2]. Renal ectopia is a congenital anomaly first described by anatomists in the 16th century. It is derived from the word “ec-topos” which in Greek means “out of place” and differentiates from ptotic kidney which has never reached its normal position in the renal fossa. The absence or the incomplete cephalad migration and rotation of the metanephric tissue and the ureteric bud in the 8th week of gestation may explain all possibilities of pelvic, iliac, abdominal, contralateral, or crossed ectopic kidney. The commonest type of renal ectopia is pelvic kidney; incidence varies from 1/2100 to 1/3000 of autopsies [3]. The pelvic kidney may be mistaken for a pelvic tumour on clinical examination [4]. The ectopic kidney is more susceptible to disease than normally positioned kidney. Because of greater risk of injuring aberrant vessels or overlying abdominal viscera and nerves, a pelvic kidney presents special treatment challenges [5]. Rotational anomalies are a rare entity that is not cited in most embryology textbooks and has important implications from the surgical point of view as in percutaneous nephrectomy and in preoperative diagnostic evaluation of kidney donors [6]. Ectopic kidneys pose a problem for any planned surgical intervention given their anomalous blood supply. Ectopic position and varied vasculature can predispose to iatrogenic trauma during interventional radiological and laparoscopic procedures and emergency operations [7]. Therefore, the knowledge of the possibility of this anatomical variation will be of help to the clinician in making a correct diagnosis and offering appropriate treatment.

## 2. Case Report

**Figure 1:**
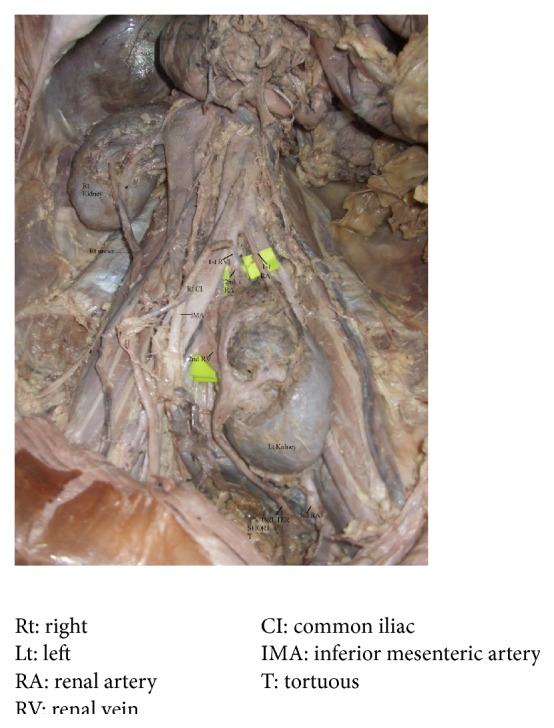
Left kidney pelvic in position, hilum anteriorly placed, open with contents exposed to the surface. Right kidney showing hilum anteriorly placed with bifid renal pelvis.

A 42-year-old male cadaver, with history of prolonged hypertension and death due to cardiac arrest, was dissected routinely. No gross variation was seen in the cadaver. After opening the abdomen a left pelvic kidney was found at the pelvic brim with its medial end in front of sacral promontory close to the bifurcation of the right common iliac artery. First its location, position, and dimensions were analysed and compared to the right kidney. The right kidney appeared normal in position. The dimensions of the left kidney were 13.5 × 6.5 × 2.5 cm, larger than the right kidney whose dimensions were 12 × 5 × 2 cm. The left ectopic kidney was lobulated. Location: the upper pole of left kidney was located at the level of sacral promontory resting over psoas major muscle and inferior pole was present at the S3-S4 intervertebral space. The hilum was anteriorly placed, open with contents exposed to the surface as seen in Figure 1. The inferior mesenteric artery was arching over the hilum. Three major calyces receiving two minor calyces were visible externally. Superior major calyx was seen reaching the upper pole while middle and inferior major calyces were seen close to the hilum all converging to form pelvis of ureter. Normal hilar relation was disturbed. At the hilum the renal pelvis was present anterior to the inferior left renal vein. The left ureter was tortuous and just 12 cm in length. Both suprarenal glands were present at the level of 12th thoracic vertebra. Blood supply: the left kidney was vascularised by three branches. The first branch arose from the bifurcation of aorta at the level of L4 vertebra; it was 4.5 cm long and descended obliquely to superior pole of kidney. The second branch was 2.5 cm and arose from the right common iliac artery at a slightly lower level (L4-L5) and was seen passing to superior pole. The third branch was 4.3 cm long arising at S5 level from left inferior vesical artery. The ectopic kidney was drained by two renal veins which drained into the right common iliac vein. The superior left renal vein accompanied the second renal artery while the inferior left renal vein emerged through the hilum.

The left suprarenal gland was supplied by left inferior phrenic artery and from direct branches from aorta. The left gonadal vein drained directly into the inferior vena cava rather than draining into the left renal vein. Left suprarenal vein drained into IVC.

The right kidney was at the normal position. However the hilum was present anteriorly and a bifid renal pelvis could be seen which was also malrotated. Its vascularisation was by a single renal artery originating from aorta. A single renal vein was seen draining into the inferior vena cava. No gross variation was seen in the cadaver.

## 3. Discussion

Cases of ectopic kidney, unilateral or bilateral, have been reported in the literature regularly (Moore and Persaud, 2008; Hollinshed, 1971) [8, 9]. Gülsün et al., 2000 [10], reported a right pelvic kidney supplied by three arteries arising from bilateral common iliac arteries and from ipsilateral internal iliac artery. Adamakis et al., 2012 [3], discovered two cases of left pelvic kidney during surgical staging of bladder carcinoma. In both cases, the left renal veins drained into IVC; the renal artery was single arising from distal part of aorta and left internal iliac artery, respectively. Both studies presented a shorter length of ureter [3]. However the present case presented different features; that is, the arterial supply was from three sources, that is, aorta, common iliac, and inferior vesical artery. The upper branches were seen supplying the superior part whereas the lower branch supplied the inferior aspect. The left renal veins drained in the right common iliac vein while left gonadal vein drained directly into the inferior vena cava. Similar to the previous case reports, the ureter was shorter in length.

There are two divergent opinions concerning the definite position of the kidney in the anatomical literature. According to the first, the kidney ascends in the retroperitoneal space during precocious ontogenic development. The renal rudiment occurs in the pelvic region, at the level of L2-L3 vertebra with the dorsal convex border and the ventral hilum touching the abdominal wall. To place itself in a definite position, the kidney undergoes ascension and rotation. Between the 6th and 9th weeks of intrauterine life, the kidney ascends to the lumber region, along the dorsal aorta. The exact mechanism is unknown. The role of an inductive substance secreted by the kidney is invoked [3]. The second opinion says that the kidney undergoes a pseudoascension caused by the fast development of the caudal extremity of the fetus [8, 11, 12]. The factors that may interfere with the renal development are teratogenic agents, genetic factors, chromosomal abnormalities, disorders in fusion mechanism of the ureteric bud and the metanephric blastema, and the medicines ingested by the mother [10]. The most frequently described cases of renal ectopia occur in males on the right side of the pelvis [13, 14]. Generally the ectopic kidney is smaller, of irregular shape and variable rotation. The kidney discussed in the present case is unilateral and has an enlarged size. The position suggests that factors have affected the renal ascension as well as the rotation process but the growth of the kidney is not affected. There is a good correlation between kidney ascension and the level of origin of the renal arteries; any anomaly in the renal artery development may delay kidney migration [8].

The following types of rotational anomalies have been identified. In* nonrotation* the renal pelvis presents itself ventrally in relation to the kidney mass. In* incomplete rotation* it presents itself ventromedially. In the more rare* reverse and excessive rotation* the renal pelvis presents itself in a position depending upon the number of degrees through which rotation has occurred [9]. This process occurs during the ascent of the kidney, which occurs between 38 and 49 days of development. Renal vascularisation occurs before definitive vascularisation. In the present case both of the kidneys have undergone incomplete rotation as renal pelvis presents ventromedially. Kidneys in ectopic (pelvic) position may go undetected in life and get noticed either in autopsy or during dissection. Often they are diagnosed for the presence of pelvic mass on phylogram. Ectopic or congenital unascended kidney has to be carefully differentiated from acquired nephroptosis where the length of ureter is normal. Symptoms may vary from none to pain: hydronephrosis, pyelonephritis, rectosigmoid fistulas, or lithiasis. Treatment is mainly based on the functional capacity of the kidney; nephrectomy is done on nonfunctional kidneys and corrective procedures are carried out forming the mainline for functional kidneys [15].

## 4. Conclusions

The ectopic kidney has clinical significance owing to its atypical location, malrotation, and vascular variations. It is vulnerable to trauma owing to its position. It may be mistaken for a pelvic tumour and removed. Urine flow or renal vascular complications can occur. A pelvic kidney presents challenges unique to its entity to a clinician including limited working space, proximity of vital structures including the great blood vessels, anomalous hilar structures, and difficulty encountered in optimal port placements. Therefore, the knowledge of the possibility of this anatomical variation will be of help to the clinician in making correct diagnosis and offering appropriate treatment.
